# In-depth qualitative interviews identified barriers and facilitators that influenced chief investigators’ use of core outcome sets in randomised controlled trials

**DOI:** 10.1016/j.jclinepi.2021.12.004

**Published:** 2022-04

**Authors:** Karen L. Hughes, Paula R Williamson, Bridget Young

**Affiliations:** a MRC-NIHR Trials Methodology Research Partnership, Department for Health Data Science, University of Liverpool, Block F Waterhouse Building, 1-5 Brownlow Street, Liverpool L69 3GL, United Kingdom; b Department of Public Health, Policy and Systems, University of Liverpool, Whelan Building, Liverpool L69 3GL, United Kingdom

**Keywords:** Core outcome set, Uptake, Clinical trials, Research waste, Interview, Qualitative research

## Abstract

**Objective:**

This study aimed to investigate barriers and facilitators to core outcome set (COS) uptake in randomized controlled trials to inform the first steps in developing interventions to improve the uptake of COS.

**Study Design and Setting:**

Semi-structured qualitative interviews with a purposive sample of United Kingdom chief investigators were audio-recorded, transcribed and analyzed thematically. Where appropriate, barriers and facilitators were mapped to components of behavior informed by the COM-B model of behavior.

**Results:**

Thirteen chief investigators were interviewed. Facilitators to uptake included: the behavior of investigators, for example, their awareness and understanding of COS; and the wider research system, for example, recommendations to use COS from funders and journals. Barriers to uptake included: the perceived characteristics of COS, for example, increasing patient burden and recommendations becoming outdated; and the COS development process, for example, not including all specialties who will use the COS.

**Conclusion:**

Based on the barriers and facilitators identified, recommendations to improve COS uptake include ensuring engagement with the research community who will use the COS, involving patients in the development of COS and ensuring COS remain up to date.

**Key findings**

• Barriers and facilitators to COS uptake included the behavior of chief investigators, the characteristics of COS and their development, and the opportunities provided by organizations in the wider health research system.
• UK chief investigators demonstrated awareness and understanding of COS which should help to facilitate their uptake.
• Recommendations to use COS from funders and journals were accepted by chief investigators and should help improve uptake of COS.
• Aspects of the COS development process, such as lack of engagement with those who will use the COS from all specialties, can hinder their uptake.

**What this adds to what is known**

• Barriers and facilitators to COS uptake were identified from the perspectives of chief investigators who have direct experience of selecting outcomes and implementing COS in randomised controlled trials.

**What is the implication, what should change now**

• COS developers can improve the uptake of COS by: engaging with the research community that will use the COS; involving patients in COS development; developing a strategy to disseminate the COS.
• COS organizations and Clinical Trialist Networks can improve the uptake of COS by: delivering education programmes; adapting guidelines to support understanding of COS; liaising with funders to support the development of COS.
• Funders can improve the uptake of COS by raising awareness and encouraging their use, and by funding work to develop measures of the recommended outcomes in the core set where suitable measures are not available.

Alt-text: Unlabelled box

## 1 Introduction

Core outcome sets (COS) are agreed, standardized sets of outcomes that should be measured and reported, as a minimum, in all clinical trials of a specific area of health or health care [1]. COS aim to address problems with the selection and reporting of outcomes in randomized controlled trials (RCTs). For example, uptake of a COS for rheumatoid arthritis demonstrates that COS can be successfully implemented [2,3]. In turn, such implementation will improve the consistency of outcomes measured across RCTs in a particular area and reduce outcome reporting bias. Involving patients in the development of COS will ensure that outcomes measured in RCTs include those important to patients [4,5].

Up to the end of 2019, 370 COS studies had been published in 32 disease categories [6] and by November 2021, 388 ongoing COS studies were registered with the COMET Initiative database (https://www.comet-initiative.org/Studies/SearchResults). These COS have the potential to reduce research waste resulting from the poor selection and reporting of outcomes in RCTs. One example of the scale of the problem comes from a systematic review of RCTs of pre-term birth prevention – 72 different primary outcomes were reported across the 103 RCTs identified between 1997 and 2011 [7]. Such inconsistencies hamper the efforts of systematic reviewers to synthesize results from RCTs, limiting the evidence available for making decisions about health care interventions. However, for this reduction in waste to be achieved it is essential that clinical trialists use COS in their studies. Whilst the COS for rheumatoid arthritis has shown successful uptake, studies of other COS have concluded that uptake needs to be improved [8,9].

The uptake of new evidence-based practices often requires behavior change by key stakeholders [10]. According to the COM-B model, whether stakeholders change their behavior depends on three factors - capability, opportunity and motivation (Table 1 provides further details). According to the Behaviour Change Wheel (BCW) [11], addressing each of three COM-B factors requires different types of intervention to overcome barriers to, or facilitate behavior change. The COM-B model and BCW provide a structure for exploring avenues for enhancing COS uptake. In this paper we draw on this structure, specifically that of the COM-B, to identify facilitators and barriers to COS uptake as perceived by trialists themselves.

**Table 1:** The COM-B model of behavior

Source of behavior and definition	Components	Examples
Capability: *being capable of performing the behavior*	Physical Psychological	Physical strength, stamina Knowledge, skills
Opportunity: *factors outside of the individual that make the behavior possible*	Physical Social	Time, location Cultural norms, social cues
Motivation: *the drive to want to perform the behavior*	Reflective Automatic	Planning, evaluating Desires, impulses

Authors of some studies assessing uptake of a COS have suggested that the characteristics of a COS may inhibit uptake, for example, by increasing patient burden [12], while others suggest that uptake is influenced by trialists’ behavior, for example, through their lack of awareness or understanding of COS [13], [14], [15], [16], [17], [18]. Exploring barriers and facilitators to uptake as perceived by trialists, can identify broader factors such as COS characteristics, or aspects of the wider health research system such as funding, that may encourage or hinder uptake.

This study aimed to inform the first steps in developing interventions to improve the uptake of COS by exploring barriers and facilitators to uptake and where appropriate mapping them to the relevant COM-B components of behavior. We conducted qualitative interviews with chief investigators (CIs) as they have direct experience of selecting outcomes and implementing COS and sampled CIs working on RCTs in any area of health research where a COS was available.

## 2 Methods

### 2.1 Study design and setting

To investigate the barriers and facilitators to the uptake of COS we conducted qualitative interviews with CIs of RCTs. Such interviews allowed the in-depth exploration of their experiences. Understanding the perspectives of CIs is likely to be important in developing interventions that would improve uptake.

### 2.2 Sampling

We purposively sampled [19] CIs who had experience of issues that might affect COS uptake, who were from a variety of clinical areas and who had different levels of experience of running RCTs to access a diversity of perspectives. We initially aimed to identify CIs of RCTs in areas where studies had found uptake of a COS to be high or low but were limited by a lack of RCTs in those clinical areas.

Individuals were eligible if they were, or had been, a CI of an RCT that was funded by the United Kingdom (UK) National Institute for Health Research Health Technology Assessment and in an area for which a relevant COS was available. The latter was important as it allowed us to explore CIs’ opinions about a specific COS that was available for use in their RCT. We determined whether a relevant COS was available by searching the Core Outcome Measures in Effectiveness Trials (COMET) Initiative database (https://www.comet-initiative.org/Studies) and reviewing whether the COS research setting, population and intervention, matched that of the RCT. Where the COS did not report a fully defined scope, for example, no intervention stated, we assumed that the COS was applicable to all interventions.

We chose a UK-based sample because the COMET Initiative is hosted in the UK and is active in promoting COS to UK-based trialists. It is therefore likely that trialists based in the UK would be aware of COS and have direct experience of the barriers and facilitators to implementing them. We chose National Institute for Health Research Health Technology Assessment CIs as this programme includes a recommendation in its guidance to applicants about searching for and using COS [8]. CIs were identified by searching the National Institute for Health Research funding and awards database (https://fundingawards.nihr.ac.uk/search). Table 2 shows the sampling frame.

**Table 2:** Sampling frame

**RCT Funder** NIHR HTA	**COS used in RCT** Yes/no
**Funding stream** Researcher-led Commissioned	**RCT Health Condition** Any with a relevant COS
**RCT start date** January 2016 or later	**COS** Any condition Published at least 1 year before RCT start date Developed for use in RCTs

CIs were sampled until data saturation had been achieved. We judged saturation to have been reached when no new codes and themes were identified during data analysis [20]. Based on recommendations for sample size in studies aiming to achieve maximum variation, we expected that between 12 and 20 interviews would be needed [21].

### 2.3 Interviews

Interviews were semi-structured, topic guided, and conversational using open-ended questions to encourage participants to freely describe their experiences [22]. The topic guide ensured that comparable topics were explored across different interviews and was informed by literature on the barriers and facilitators to clinicians’ uptake of clinical guidelines [23], [24], [25] and by the COM-B model (11) (Appendix 1 for the topic guide). Additional questions were asked based on participant's responses allowing the exploration of topics that arose during the interviews. Before interviews we sent participants a publication describing the COS that was relevant to their clinical area and invited them to read this so we could explore their views of the COS during the interview. If the participant had not read the publication the interviewer gave a brief summary during the interview.

Interviews were via telephone or Zoom video-conference (https://zoom.us/), except for one which was carried out face-to-face at the request of a participant. All interviews were carried out by KH and were audio recorded, transcribed verbatim and anonymized. KH had attended training for conducting and analyzing qualitative interviews with the Health Experiences Research Group, University of Oxford.

Participants provided signed consent via email prior to the interview or gave verbal consent which was audio recorded. The University of Liverpool Health and Life Sciences Research Ethics Committee (Psychology, Health and Society) gave ethical approval for the study on 29/04/2019 (reference 4840).

### 2.4 Analysis

Our approach to analyzing CIs’ accounts was interpretive – we treated these as offering insights about their perspectives, rather than as providing direct access to their perspectives. Informed by CIs’ accounts and the COM-B model, our identification of barriers and facilitators to COS uptake was pragmatist.

We analyzed the interviews thematically [26] and alongside data collection. First, we approached the data inductively by listening to audio recordings of the interviews and reading transcripts along with field notes in which initial impressions had been recorded. We analyzed transcripts line by line to identify codes and categories. We then carried out deductive analysis informed by the COM-B model of behavior as a coding framework, and assigning codes and categories to the relevant components of the COM-B model. All codes identified could be categorized within the coding framework that was informed by the COM-B model. Supplementary File 1 shows how the codes mapped to the dimensions of the COM-B model.

Coding was carried out by KH and periodically discussed with PW and BY, who also read most transcripts. NVivo 12 Pro was used as a data management tool (https://www.qsrinternational.com/).

It is important to be reflexive in qualitative research and we recognized that the authors’ perceptions and beliefs will have played a role in shaping the study and interpretation of the data [27], particularly as all authors have a longstanding interest in COS. Moreover, PW is chair of the COMET Initiative which promotes the development and uptake of COS. BY is co-chair of the COMET People and Patient Participation Involvement and Engagement (PoPPIE) group and KH was a PhD student with links to the COMET Initiative. We were therefore mindful of how CIs might perceive us and how this might influence the interviews. Specifically, to ensure transparency whilst also supporting CIs in speaking candidly, KH emphasized her position as a PhD student looking to learn from their experiences, rather than as representing the COMET Initiative. This was outlined in the invitation email, study information sheet and KH also reiterated this at the start of the interviews. KH also continually reviewed her approach to interviewing to ensure questions were neutral and convey that she was nonjudgementally exploring CIs’ perspectives on COS whatever these were.

Quality of the conduct and reporting of the study was guided by the Standards for Reporting Qualitative Research (SRQR) [28].

## 3 Results

We invited 19 CIs for interview, and KH interviewed 13 CIs between July 2019 and February 2021. Start dates of the RCTs ranged from May 2016 to April 2020 and were assigned to the following NIHR-defined health categories: Respiratory (1), Musculoskeletal (2), Cancer (1), Reproductive Health and Childbirth (3), Injuries and Accidents (3), Skin (2) and Generic Health Relevance (1). Further details of the RCTs and COS are presented in Table 3.

**Table 3:** Details of COS and RCTs

Participant ID	Funding stream	No. years COS published before start date of RCT	Used COS (Yes, no, partially)
CI1	Researcher-led	5	Yes
CI2	Researcher-led	4	No
CI3	Researcher-led	11	Yes
CI4	Researcher-led	3	Yes
CI5	Researcher-led	11	Yes
CI6	Researcher-led	1	Partially
CI7	Researcher-led	<1 (involved in development)	Yes
CI8	Researcher-led	15	No
CI9	Researcher-led	1	Partially
CI10	Researcher-led	10	Partially
CI11	Commissioned	1	No
CI12	Commissioned	2	Yes
CI13	Commissioned	1	Yes

Identification of barriers and facilitators to uptake of COS was informed by COM-B components: capability (psychological), opportunity (social) and motivation (automatic and reflective).

### 3.1 Capability

CIs raised issues linked to psychological capability, that is, knowledge and skills needed to implement COS, as influences on uptake of COS.

With one exception (CI8), all CIs reported having previously heard of COS before being invited to interview. Indeed, CIs were not just aware of COS - several described being involved in COS development (CI5). CIs also spoke of how dissemination by COS developers and external organisations, for example, funders, and colleagues had raised their awareness of COS.

They were also aware of problems that COS aim to address, such as inconsistency of outcomes measured across RCTs:

*“You have got two trials that have reported this and one that has reported that and three…you know, it's all bitsy when you try to put it together.” (CI7)*

*“There's the knock-on of being more consistent, so that would help with evidence synthesis and help reduce waste.” (CI6).*

While inconsistency of outcomes was a recurring theme, one CI pointed out that this was not the case in their clinical area as: “*There are very few outcomes” (CI10),* and as a result RCTs in this area were measuring consistent outcomes.

CIs also indicated their understanding of how to apply COS and that using COS does not preclude the measurement of other outcomes. For example, CI6 referred to the importance of COS developers making it clear that the use of COS did not preclude the measurement of other outcomes that may be important to a particular RCT: *“if people view it in the way that they're the only ones [outcomes] that should be measured.” (CI6)*

Whilst CIs indicated their understanding that all outcomes recommended by a COS should be measured, one suggested that: *“you have to be given the right to pick and choose” (CI10),* depending on what outcomes are suitable for the RCT and not *“pick it off the shelf and use it without it being checked” (CI3)*. This included CIs critiquing the methods used to develop the COS and ensuring that the COS was up to date.

### 3.2 Opportunity

Linked to social opportunity, such as the cultural norms and social cues which influence behavior, CIs spoke about receiving encouragement to use a COS from organizations such as journals and funders of RCTs:

*“We had some feedback from the funders that we should consider the core outcome set.” (CI2)*

*“And there has been buy-in from the journals, so I think we wanted to be- to make sure that our research kept pace with it.” (CI12)*

One CI spoke about the prospect of COS becoming the ‘norm’ as helping to facilitate uptake:

*“Yes, if it became a more widely accepted thing, that would probably influence other people more.” (CI6)*

Linked to the earlier point about raising awareness through peers, CI6 commented that it was important for the research community to also provide social opportunity to facilitate use COS:

*“The community has a responsibility as well. People, who are aware of it, making others in their network aware of it.” (CI6)*

### 3.3 Motivation

#### 3.3.1 Automatic motivation

CIs spoke about desires and impulses that may affect their uptake of COS. Under the COM-B model these relate to automatic motivation. These included “territory” and “moral obligation.”

Territory was spoken about as a barrier to COS uptake where CIs may have developed their own outcome measurement instruments that had not been included in the COS. CIs also commented that other CIs would want to control what outcomes were measured in their RCT and might not accept decisions they had not been involved in:

“There's territory here … the problem is when groups get together and try and decide them, is that inevitably *people that have developed measures that are then not selected can obviously feel a bit put out” (CI1)*

“Y*ou get one particular person who's like, “I'm not going to have anything to do with that group,” and they go off on a tangent on their own.” (CI3)*

One CI suggested that in these instances CIs have a moral obligation to put territorial considerations aside so that the benefits of COS can be realized:

*“The other responsibility trialists have as well is, as far as possible, trying to respect the recommendations and including them in your trial even if it's not your favourite outcome measure, because I think there's a bit of a moral obligation there” (CI1)*

#### 3.3.2 Reflective motivation

When CIs shared their evaluations of COS and the COS development processes, they focused on barriers related to COS, the drawbacks of using COS and issues with the COS development process, rather than barriers linked to their own behavior.

#### 3.3.3 The drawbacks of COS

Patient burden was a recurring theme in CIs’ accounts of the drawbacks of COS, with several believing that using COS could lead to an increase in the number of outcomes to be measured. CIs were particularly concerned that COS might increase the number of outcome instruments that patients were asked to complete, exacerbate repetition across outcome measurement instruments and diminish the relevance of outcomes to patients:

*“It's a real issue in core outcome sets, that you end up with a list that is so long that you struggle with burden” (CI9)*

*“Participants certainly don't like it if they're filling in a 50-item questionnaire and feel the first 20 items are repetitive …” (CI1)*

*“There's the meaning for the patient as well. If they're answering questions they don't feel have any meaning, they're less incentivised to complete it.” (CI4)*

CIs were more comfortable with an increased number of outcomes if the collection of these did not impact upon patients:

*“I don't think it's a big burden on patients, because most of it comes from records.” (CI7)*

They were also concerned about COS becoming outdated as new technologies and outcome measurement instruments were developed. As CI1 pointed out, *“there's always a possibility that what's recommended now won't be recommended in five- or ten-years’ time.”* Whilst CIs spoke about the importance of updating COS, CI13 pointed out some of the difficulties with this process. This included deciding when an update would be appropriate and who should be responsible for updating the COS:

*“I mean, okay, somebody has developed a core outcome set and it's out there. Then another group comes along and says, “Well this is all outdated, I want to do another core outcome set.” … Who decides that a core outcome set I've developed is now obsolete?” (CI13)*

As a potential solution, CI13 suggested that a structure should be put in place to regulate the updating of COS.

#### 3.3.4 COS development process

Some CIs expressed concerns about COS being developed *“because there is a gap in the market” (CI4),* without first investigating whether a COS is needed. CI4 suggested more consideration of the need for and suitability of a COS prior to initiating the development process:

*“What needs to happen is probably there needs to be as much attention paid to the topic itself before anything is done, whether it's appropriate to have a core outcome set for that particular thing they're trying to do it for.” (CI4)*

Echoing findings described earlier in relation to territory, a common theme throughout the interviews was the lack of engagement that CIs felt with the COS development process which they felt could lead to poor uptake:

*“There's a thing that a lot of [clinicians in the specialty] feel that this is developed by people who don't actually- who aren't involved in our world. And I think there's a thing about engagement here, that is a real problem.” (CI9)*

CI9 went on to explain that the absence of a representative for their role and specialty in the development of the COS could lead to CIs disengaging and not using the COS.

Alongside COS recommending *what* outcomes to measure, some CIs said they had hoped the recommendations would include *how* to measure these outcomes. Some were concerned about achieving consistency not just in outcomes but in measurement instruments too:

*“It's all well and good having, say, health-related quality of life in all studies, but if everyone measures it a different way then you might be left with the same problem of not necessarily being able to combine data from different quality of life tools.” (CI6)*

Linked to these concerns, CIs spoke about COS development as presenting an opportunity to improve the quality of outcome measurement instruments and one CI expressed disappointment that a particular COS had not led to such improvements:

*“I looked more at the overall domains and the fact that they couldn't tell me all the measures. I just thought, right, this is not really helping me very much, when are you going to do some more?” (CI11)*

## 4 Discussion

### 4.1 Main findings

Our study set out to identify barriers and facilitators to COS uptake informed by the perspectives of CIs of RCTs and the COM-B framework linked to the capability, opportunity and motivation of CIs, as a first step towards developing interventions to improve the uptake of COS. In Table 4 we suggest actions, informed by the findings, to overcome these barriers and facilitate COS uptake.

**Table 4:** Summary of suggested actions to improve uptake

Who	Action	Intention that will improve uptake of COS	COM-B
COS developers	Determine if a COS is needed prior to starting the development process	Ensures necessity and suitability of COS for the clinical area	Reflective motivation
	Develop a dissemination plan including website, publication, social media, conferences	Raise awareness of COS among clinical trialists	Psychological capability
	Invite clinical trialists from all clinical roles and specialties relevant to the scope of the COS to be involved in its development	Raise awareness of COS among clinical trialists Increase engagement with clinical trialists Ensure outcomes important to clinical trialists are included in the COS	Psychological capability Reflective motivation Reflective motivation
	Involve patients in the development of COS	Ensure outcomes important to patients are included in the COS	Reflective motivation
	Consider the impact of collecting the outcomes on patients	Reduce patient burden of COS	Reflective motivation
	Recommend outcome measurement instruments as well as outcomes	Improve consistency in how outcomes are measured Tackle uncertainty around which outcome measurement instruments to use Identify improvements needed to outcome measurement instruments	Reflective motivation Reflective motivation Reflective motivation
	Engage with individuals in the research community who are committed to the longevity of the COS	Increase likelihood of COS being completed and maintained	Reflective motivation
COS organizations, Clinical Trialist Networks	Deliver information about COS in educational programmes	Raise awareness of COS among clinical trialists Increase knowledge of COS and their purpose Facilitate understanding of how to apply COS	Psychological capability Psychological capability Psychological capability
	Adapt the general guidelines on assessing the relevance and applicability of COS as appropriate	Assist CIs in assessing the relevance and applicability of COS for a particular RCT	Psychological capability
	Liaise with funders over lack of funding to fully develop COS	Facilitate a source of funding to enable the completion of COS	Reflective motivation
	Provide structure to the process of updating COS	Encourage regular reviews and appropriate updates of COS	Reflective motivation
Funders	Recommend the use of COS in application forms	Raise awareness of COS among clinical trialists	Psychological capability
	Feedback to applicants about COS	Support clinical trialists to implement COS	Social opportunity

Regarding the psychological capability that enabled CIs to use COS, this encompassed how CIs became aware of COS, their understanding of the need for them, and knowing how to apply them. CIs described several ways in which they had learned of COS, for example, websites, publications, social media and conferences, generalizing previous findings about dissemination in studies focusing on specific COS [29,30]. We recommend that COS developers plan their dissemination strategy and consider methods to maximize awareness of the COS. We also suggest that understanding how to apply COS could be further improved by the production of guidelines on how to assess the applicability of a COS for a particular RCT. The COMET Initiative delivers training workshops and is developing further guidance to assist CIs in assessing the relevance and applicability of COS.

In addition to facilitators relating to capability, CIs discussed how COS uptake was linked to social opportunities provided by the wider health research system. Specifically, they believed funders and journals could facilitate implementation of COS by raising awareness and encouraging their use. As noted previously [8], funding boards could also facilitate COS uptake by informing CIs where a COS is available but this has been omitted from a funding application. Furthermore, the inclusion of a check list with funding applications could encourage applicants to report their search for a COS or explain why a search had not been carried out. Similarly, journals could request that authors justify their use of a COS (or not) as part of the submission process in the same way that authors are asked to specify which reporting guideline they have followed.

A barrier to uptake linked to reflective motivation, as indicated by CIs’ evaluations of the COS development process, was a perception that COS were being developed because one does not exist rather than there being a need for that COS. To contextualize this, in the case of one CI who raised this issue, the authors of the relevant COS in the CI's area had outlined their aim to develop a minimum set of outcomes but did not report whether there were inconsistencies in the outcomes being measured across different RCTs in the clinical area. Before starting the COS development process, it would be prudent for developers to carry out investigations into whether a COS is needed, for example, by engaging with clinical trialists and systematic reviewers and assessing the state of outcomes in the area.

A further concern of CIs linked to reflective motivation was patient burden. The COS relevant to some of the CIs who discussed this barrier recommended six and seven outcomes suggesting the concerns of these CIs were not strongly founded and interventions to improve uptake might focus on enhancing CIs’ knowledge of COS in their areas and their motivation to use these. However, another COS recommended 17 outcomes suggesting the concerns of the CI working in this area were more strongly founded and pointing to the importance of developers considering the demands that COS place on patients. One CI noted that most of the outcomes for the COS they used were collected routinely and did not increase patient burden. This move towards core outcomes also being of relevance clinically could mean the core outcomes would be measured routinely thus streamlining the process of doing research.

Some CIs or their co-investigators had been involved in COS development. Involving end users in COS development, as recommended by the COS-STAD minimum standards for COS development [31], increases the relevance of the outcomes included, as well as promoting user engagement. Under reflective motivation some CIs described reluctance to use a COS if their role and clinical specialty had not been represented in its development. In some cases where this was raised, the COS publication reported limited stakeholder involvement, such as clinical experts only, suggesting these CIs’ concerns were founded. This points to the importance of ensuring that all clinical roles and relevant specialties are represented throughout the development process. Engaging with individuals in the research community who are committed to the longevity of the COS would also increase the likelihood of the COS being maintained and updated in the future. However, for other COS publications indicated that a broad range of stakeholders had been involved in the development process, suggesting this was more of a perceived barrier than one arising from how the COS had actually been developed. This again indicates that interventions focused on enhancing CIs’ knowledge of COS in their areas and motivation to use these are also needed.

CIs highlighted the need for high-quality COS. This included ensuring the outcomes recommended by COS are meaningful to patients. As recommended by COS-STAD [31], it is important that patients are involved in the development of COS and input into what outcomes and measurement instruments are recommended to improve the quality of COS.

An absence of recommendations for how to measure the outcomes included in a COS presented a barrier for some CIs. It has been reported that of 337 published COS studies, 118 (35%) had completed both the *what* and *how* stages [32]. CIs suggested that it was important that COS recommend outcome measurement instruments to ensure consistency in how the outcomes were being measured. Some CIs spoke about a lack of quality outcome measurement instruments available for their clinical area and their hopes that this absence would be addressed by the development of the COS. Where quality outcome measurement instruments are lacking, COS developers should take the opportunity to recommend the development of new instruments as part of a research agenda. Funders often fund COS development and could facilitate the completion of both stages of the COS by committing to funding the development of the *how* to measure as well as *what* to measure.

A facilitator to uptake identified during the interviews, linked to social opportunity, was COS ‘becoming the norm’. This aligns with previous research exploring the implementation of COS in hemodialysis, which identified culture change as important to uptake [33].

This study has identified barriers and facilitators to the uptake of COS in RCTs, but COS are also recommended for use in systematic reviews [34]. Surveys of coordinating editors of Cochrane Review Groups found support for the use of COS in systematic reviews [35,36]. However, studies that have assessed uptake of COS in systematic reviews have reported variable uptake of individual outcomes recommended by the COS [15,37]. Future qualitative work to explore the barriers and facilitators to uptake of COS in systematic reviews may be helpful, as it cannot be assumed these will be the same as those that affect trialists’ use of COS.

### 4.2 Strengths and limitations

Qualitative interviews and analysis provided insights into the barriers and facilitators that influence COS uptake from the perspectives of CIs who have experience of choosing outcomes for an RCT. Open-ended-questions encouraged participants to share their experiences and further explore unanticipated topics. Using the COM-B model has informed the identification and classification of barriers, as a first step towards developing theoretically informed interventions to improve COS uptake.

Participation in the interviews was limited to CIs based in the UK and whose RCTs had been funded by the National Institute for Health Research Health Technology Assessment. These CIs were chosen as they are more likely to be aware of COS due to funder endorsement and have direct experience of the barriers and facilitators to implementing COS. We were therefore unable to explore lack of awareness as a barrier due to our sampling approach. It is important to recognize that in countries where there is less awareness of COS, the barriers and facilitators to uptake may differ. Further work is needed to investigate barriers and facilitators to uptake of COS in settings beyond those in this study.

While the sample size for the study was relatively small, we continued to sample until data saturation had been reached. We judged that saturation had been reached after ten interviews, but carried out three more interviews to check this.

### 4.3 Conclusion

Facilitators to the uptake of COS by CIs of RCTs were identified in relation to capability and opportunity. Developing guidelines to assist CIs in assessing the applicability of a COS and encouraging funders and journals to endorse COS could build on these facilitators. Barriers to COS uptake also were identified in relation to motivation. Some of these barriers could be addressed by including patients in COS development and recommending outcome measurement instruments as well as outcomes. Further work is needed to identify the most appropriate interventions, informed by the BCW where appropriate, to improve uptake of COS.

## Author contribution

Karen L. Hughes: Conceptualization, Methodology, Data curation, Formal analysis, Investigation, Writing - original draft. Paula R Williamson: Conceptualization, Methodology, Writing - review & editing, Supervision. Bridget Young: Conceptualization, Methodology, Writing - review & editing, Supervision.

## Appendix Supplementary materials

Image, application 1

Image, application 2
