# Supplementary material for: In-depth qualitative interviews identified barriers and facilitators that influenced chief investigators’ use of core outcome sets in randomised controlled trials
Source: J Clin Epidemiol. 2022 Apr;144:111–20. doi: 10.1016/j.jclinepi.2021.12.004 (PMC9094758; doi:10.1016/j.jclinepi.2021.12.004)
Supplement: Supplementary file 1 [file mmc1.docx]

## Appendix 1: Interview topic guide

**Interview topic guide**

**Outcomes**

***Selecting outcomes for the trial***

- Walk me through how you and the team decided on which outcomes to measure in [trial name]
- Which members of the trial team were involved in choosing the outcomes for the trial?
- What or who influenced your choice of outcomes?

Prompt - What sources of information informed your decisions? Who did you ask?

***Awareness of problems with outcomes***

- Tell me about any problems you see with the outcomes that are measured in trials in your clinical area?

Prompt – are there differences in the outcomes measured across trials in your clinical area? Are the outcomes relevant to patients and healthcare professionals (HCP)?

- What is the impact of these problems?
- When you were deciding on outcomes for [trial name] did you consider the possibility of the findings eventually being combined with those from other trials. Did you take any steps to make sure that your results could be combined? Can you describe what steps you took?
- How did you make sure that your chosen outcomes were relevant to HCP and patients?

**Core outcome sets**

Before the interview I sent you a paper about a core outcome set that is linked to your clinical area.

***Knowledge/awareness of COS***

- Were you aware of COS before you read the paper? If yes, how did you find out about COS?
- [If interviewee didn’t know about COS, or that particular COS], how would you choose to be notified about the COS? Who should notify you/disseminate the COS?
- Can you tell me about what you see as the reasons for developing COS? What are the overall goals?
- Are the goals achievable?
- What are the drawbacks of using COS?

***Confidence in COS***

- What did you think of the methods used to develop the COS in the paper I sent to you?
- Who should be involved in the development process?
- In what ways would the methods used and stakeholders involved in developing a COS influence your decision to use it?
- What part should trialists play in the development of COS?

Prompt - At what point should they be involved? Would you get involved in COS development?

- What key things should developers consider when deciding on which outcomes will be included in the COS?

Prompt – HCP/patient burden and current available measures

- Was it clear from the COS publication which outcomes are in the COS? How did the outcomes in the COS compare to the outcomes you chose? Do you agree with the COS?
- Is there any more information that you would like see provided to enable you to use the COS?

***Using COS***

- Did you use a COS in your trial? What influenced your decision to use or not use a COS?
- If you weren’t aware of COS before, would you use one now? What would influence your decision?
- If more than one COS exists for your area how would you decide which to use? What information would you need to judge which was most suitable?
- If a funder recommended that applicants use a COS, would it influence your decision to use COS?

Or a clinical trials registry?

Or a journal?

- Would it be helpful to be reminded about COS when designing your trial? Who should do that? e.g. funder
- What other things would motivate you to use a COS?

***Closing interview***

- That’s the end of my questions. Is there anything else that is important to you that we haven’t talked about?
- Is there anything else you’d like to say?

Close interview
