# Supplementary figures and images for: In-depth qualitative interviews identified barriers and facilitators that influenced chief investigators’ use of core outcome sets in randomised controlled trials
Source: J Clin Epidemiol. 2022 Apr;144:111–20. doi: 10.1016/j.jclinepi.2021.12.004 (PMC9094758; doi:10.1016/j.jclinepi.2021.12.004)

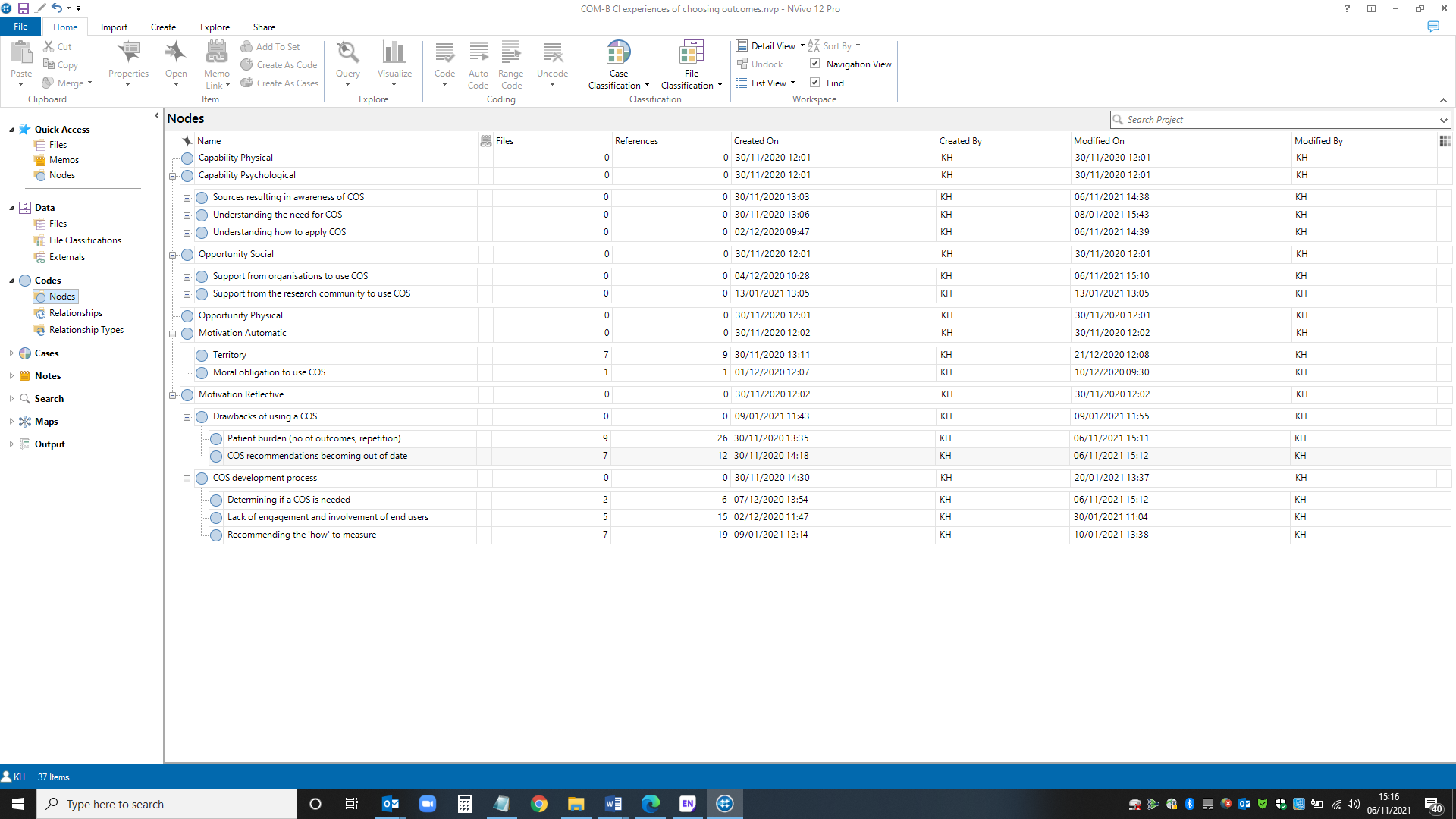

Supplement: Supplementary file 2 [file mmc2.docx]
